# Supplementary material for: Cyclin CLB2 mRNA localization and protein synthesis link cell cycle progression to bud growth
Source: Nat Commun. 2025 Nov 26;16:11654. doi: 10.1038/s41467-025-66623-w (PMC12749197; doi:10.1038/s41467-025-66623-w)

Supplementary information

Cyclin CLB2 mRNA localization and protein synthesis link cell cycle progression to bud growth

Anna Maekiniemi[1,‡] , Philipp Savakis[2,‡], Kelly van Rossum[2], Jacky L. Snoep[3,4], Markus Seiler[5], David D. van Niekerk[3], Kathi Zarnack[5,6], Robert H. Singer[1], Evelina Tutucci[1,2,*]

[1] Cell Biology, Albert Einstein College of Medicine, 1300 Morris Park Avenue, 10461 Bronx, NY, USA

[2] A-LIFE Department, Systems Biology Section, Amsterdam Institute of Molecular and Life Sciences (AIMMS), Vrije Universiteit Amsterdam, De Boelelaan 1108, NL-1081HZ Amsterdam, The Netherlands

[3] Department of Biochemistry, University of Stellenbosch, Stellenbosch 7600, South Africa

[4] Department of Molecular Cell Biology, Vrije Universiteit Amsterdam, De Boelelaan 1085, 1081HV, Amsterdam, The Netherlands

[5] Buchmann Institute for Molecular Life Sciences (BMLS) & Faculty of Biological Sciences, Goethe University Frankfurt, Max-von-Laue-Str. 15, 60438 Frankfurt, Germany

[6] Theodor Boveri Institute, Julius Maximilians University Würzburg, Biocenter, Am Hubland, 97074 Würzburg, Germany

‡ Equal contributions.

*Corresponding author: Evelina Tutucci evelina.tutucci@vu.nl

Supplementary Figures

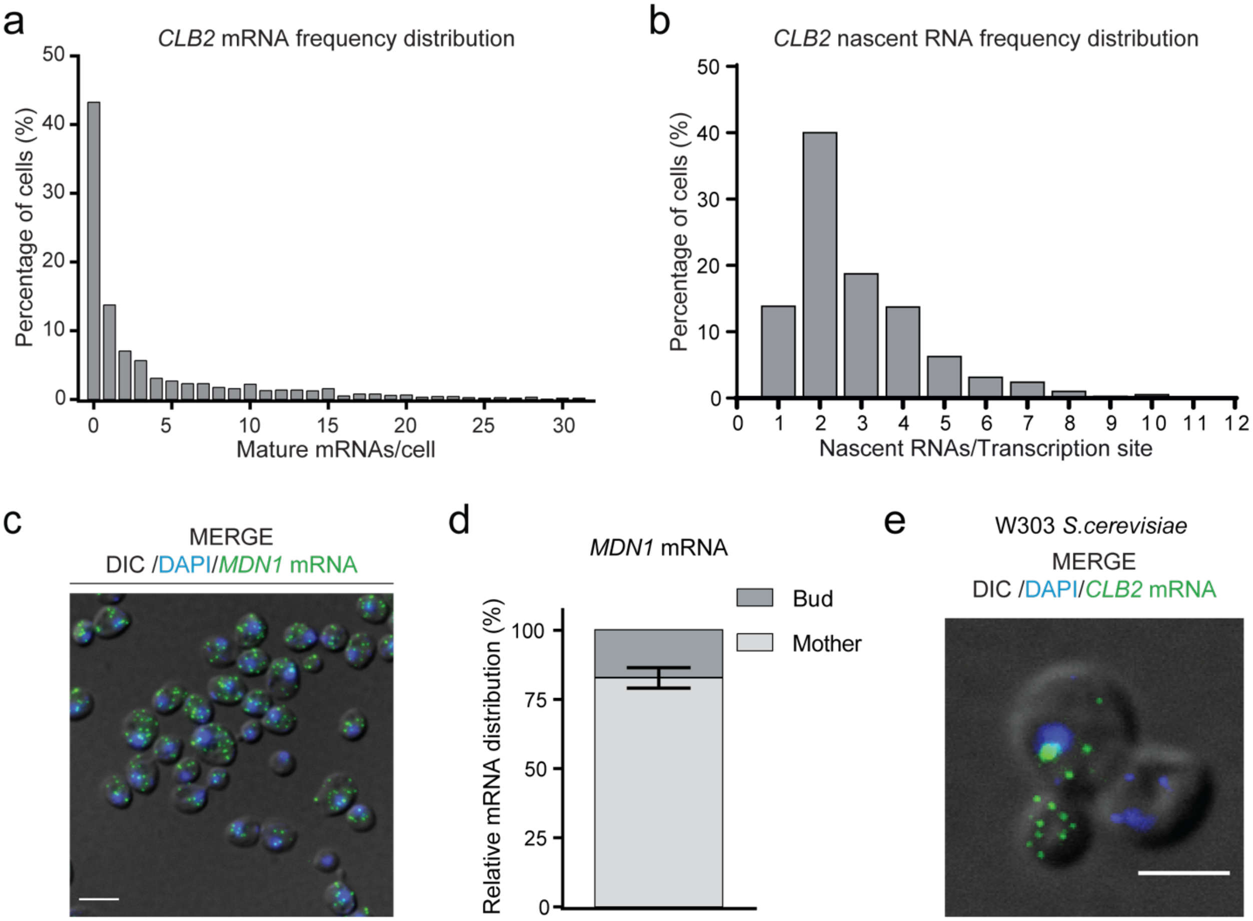

Supplementary Figure 1. *CLB2* mRNAs are localized also in a different *S. cerevisiae* background unlike a control mRNA.
(a) Quantification of *CLB2* mRNA smFISH shown in Fig. 1b reported as relative frequency distribution of mature mRNAs per cell. Data from two replicates (n=2083). **(b)** Quantification of *CLB2* nascent RNAs at transcriptions sites (TS) from smFISH shown in Fig. 1b reported as relative frequency distribution of nascent RNAs per TS. Data from two replicates (n=2083). **(c)** MERGE, maximal projection of *MDN1* smFISH (green), and DAPI (blue) merged to a single differential interference contrast (DIC) picture (grey). Scale bar 5 μm. **(d)** Relative bud vs mother distribution of *MDN1* mRNA in budded cells based on the smFISH data shown in (c). Data from two replicates (n=2011; 82.9±3.7, mean ± SD). **(e)** smFISH in the *S. cerevisiae* background W303. MERGE, maximal projection of *CLB2* mRNA smFISH (green) and DAPI (blue) merged to a single DIC picture (grey). Scale bar 3 μm. Source data are provided as a Source Data file.

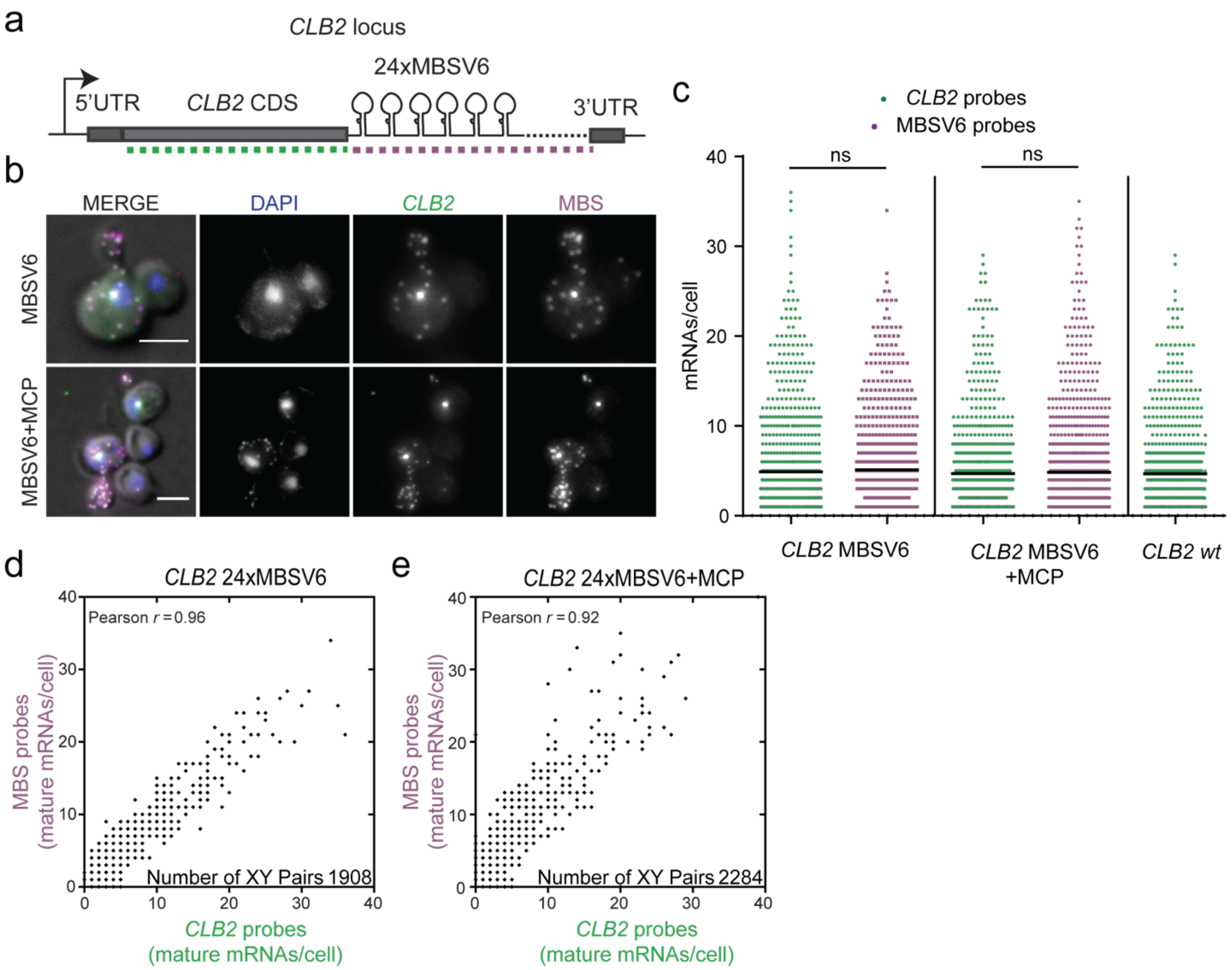

Supplementary Figure 2. *CLB2* tagging with the MBSV6 reporter recapitulates *CLB2* mRNA expression.
Schematic of *CLB2* locus endogenously tagged with 24xMBSV6 inserted in the 3' UTR right after the STOP codon. Dotted lines represent smFISH probe positions targeting the CDS (green) or MBS sequences (magenta). **(b)** Two-color smFISH for cells expressing tagged *CLB2* mRNAs. Top panels (MBSV6): cells expressing the control vector (YcpLac111). Bottom panels (MBSV6+MCP): cells expressing MCP (YcpLac111 CYC1p-MCP-NLS-2xyeGFP). MERGE, maximal projections of *CLB2* CDS smFISH (green), MBS (magenta), and DAPI (blue) merged to a single DIC picture (grey). Individual fluorescence channels are shown in grayscale. Scale bars 3 µm. **(c)** Quantification of smFISH shown in (b) as well as untagged WT cells, with CDS probes (green plots) or MBS probes (magenta) reported as distribution of mature mRNAs per cell. Mean of three biological replicates, (*CLB2* MBSV6: CLB2 probes n= 864, mean ± SD 4.9 ± 5.9 mRNA/cell; MBSV6 probes n=771, mean ± SD 5.4 ± 6.1 mRNA/cell; *CLB2* MBSV6+MCP: *CLB2* probes n = 723, mean ± SD 4.5± 5.0 mRNA/cell, MBSV6 probes n=977, mean ± SD 4.9±6.1 mRNA/cell; WT cells: *CLB2* probes n = 791, mean ± SD 5.1±5.1 mRNA/cell). Statistical differences were calculated by non-parametric Mann-Whitney test. **(d)** and **(e)** Correlation between the number of single CDS and MBSV6 molecules per cell in presence or absence of MCP. Pearson *r* values calculated by combining two independent experiments (n=1908 and n=2284, respectively). Source data are provided as a Source Data file.

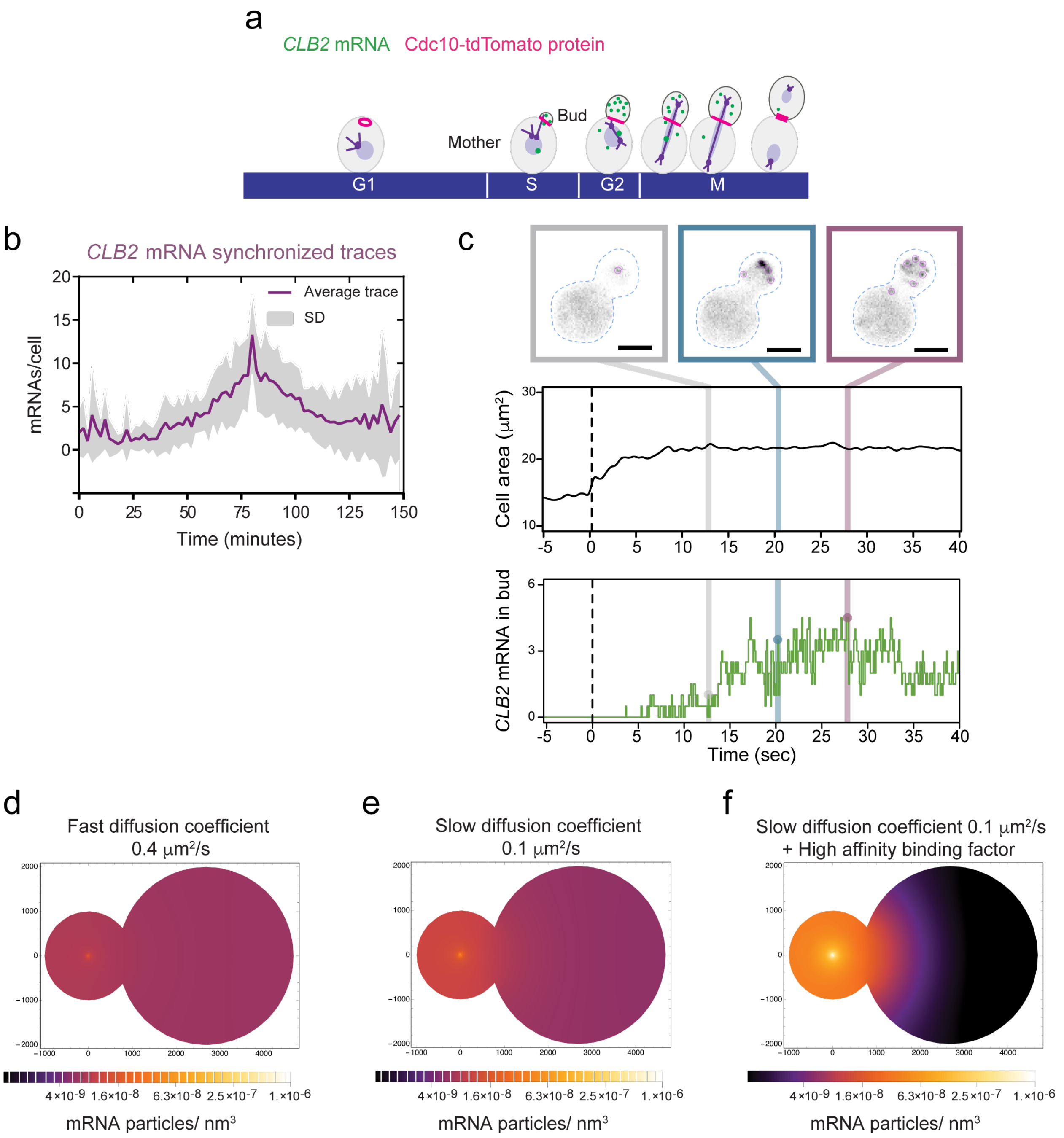

Supplementary Figure 3. *CLB2* mRNA imaging throughout the cell cycle reveals a rapid mRNA accumulation and localization to the bud and mathematical modelling predicts the existence of mRNA anchoring factors in the bud.
(a) Schematic of *CLB2* localization during the cell cycle. The *CLB2* mRNA (green), the bud neck protein Cdc10 is tagged with tdTomato (magenta). **(b)** Average number of *CLB2* mRNA per cell tagged with 24xMBSV6 monitored over time (purple line), grey represents the standard deviation (SD). **(c) Top.** Snap shots from live cell imaging of *CLB2* mRNA endogenously tagged with 24xMBSV6. Approximate cell outline identified from fluorescent background (dashed blue line). A single Z plane was acquired every 100 ms. Scale bars 3 μm. **Middle.** Average cell area monitored over time in live cells (n=3). A single Z plane was acquired every 100 ms. Pink, purple and green

lines represent time points at which the snapshots on top were taken. **Bottom.** Average number of bud localized mRNAs per cell over time as monitored from 3 single cells. Pink, purple and green lines represent the time points at which the snapshots on top were taken. **(d-f)** Simulation of localization of mRNA particles per nm^3 assuming **(d)** fast diffusion coefficient of 0.4 $\mu m^2/s$, **(e)** slow diffusion coefficient of 0.1 $\mu m^2/s$ and **(f)** slow diffusion coefficient of 0.1 $\mu m^2/s$ in combination with a high-affinity binding factor. Source data are provided as a Source Data file.

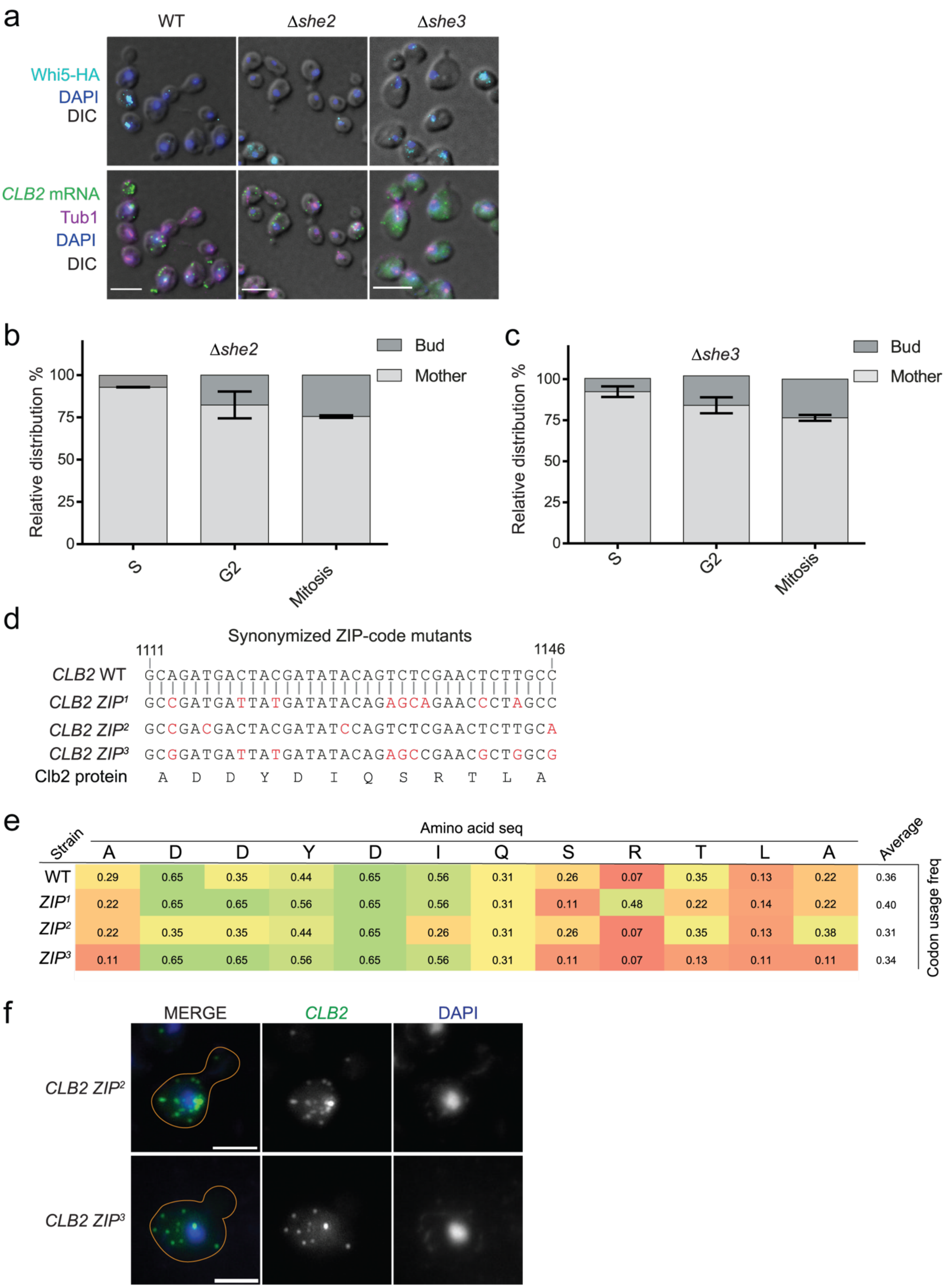

a
WT
Δshe2
Δshe3
Whi5-HA
DAPI
DIC
CLB2 mRNA
Tub1
DAPI
DIC
b
Δshe2
Bud
Mother
Relative distribution %
100
75
50
25
0
S
G2
Mitosis
c
Δshe3
Bud
Mother
Relative distribution %
100
75
50
25
0
S
G2
Mitosis
d
Synonymized ZIP-code mutants
1111
1146
CLB2 WT GCAGATGACTACGATATACAGTCTCGAACTCTTGCC
CLB2 ZIP1 GCCGATGATTATGATATACAGAGCAGAACCCTAGCC
CLB2 ZIP2 GCCGACGACTACGATATCCAGTCTCGAACTCTTGCA
CLB2 ZIP3 GCGGATGATTATGATATACAGAGCCGAACGCTGGCG
Clb2 protein A D D Y D I Q S R T L A
e
Amino acid seq
Strain A D D Y D I Q S R T L A Average
WT 0.29 0.65 0.35 0.44 0.65 0.56 0.31 0.26 0.07 0.35 0.13 0.22 0.36
ZIP1 0.22 0.65 0.65 0.56 0.65 0.56 0.31 0.11 0.48 0.22 0.14 0.22 0.40
ZIP2 0.22 0.35 0.35 0.44 0.65 0.26 0.31 0.26 0.07 0.35 0.13 0.38 0.31
ZIP3 0.11 0.65 0.65 0.56 0.65 0.56 0.31 0.11 0.07 0.13 0.11 0.11 0.34
Codon usage freq
f
MERGE
CLB2
DAPI
CLB2 ZIP2
CLB2 ZIP3

Supplementary Figure 4. *CLB2* is not bud localized in the *Δ*she mutants and alternative ZIP code mutants.

(a) smFISH- IF in WT, *Δshe2* and *Δshe3* cells: Top panels, MERGE, maximal projections of IF anti-HA (Whi5-HA, cyan) and DAPI (blue) merged to a single DIC picture (grey). Bottom panels, MERGE, maximal projections of *CLB2* mRNA smFISH (green), anti-tubulin IF (magenta) and DAPI (blue) merged to a single DIC picture (grey). Scale bars 5 µm. **(b)** Relative bud vs mother distribution of the *CLB2* mRNA in *Δshe2* budded cells based on the smFISH-IF data shown in (a) and Fig. 2a. (S phase= 92.9±0.2, G2 phase= 82.4±7.9, M phase= 75.5±0.7; mean ± SD). **(c)** Relative bud vs mother distribution of the *CLB2* mRNA in *Δshe3* budded cells based on the smFISH-IF data shown in (a) and Fig. 2a. (S phase= 92.44±3.3, G2 phase= 84.0±4.6, M phase= 76.4±1.8; mean ± SD) (S phase= 58.4±5.5, G2 phase= 34.6±0.9, M phase= 34.4±7.2; mean ± SD). **(d)** Synonymized ZIP-mutant variants (ZIP^1, ZIP^2 and ZIP^3) comparison to *CLB2* WT (top sequence). Mutated nucleotides are indicated in red. Below is the corresponding Clb2 protein amino acid sequence, which is identical for both the WT and synonymized strains. **(e)** Table summarizing the codon usage frequency for the sequences of the three ZIP code variants, ZIP^1, ZIP^2 and ZIP^3 compared to the WT ZIP code. **(f)** smFISH in the ZIP code mutant strains ZIP^2 and ZIP^3. MERGE, maximal projections of *CLB2* mRNA smFISH (green) and DAPI (blue). Individual fluorescence channels are shown in grayscale. Scale bars 3 µm. Source data are provided as a Source Data file.

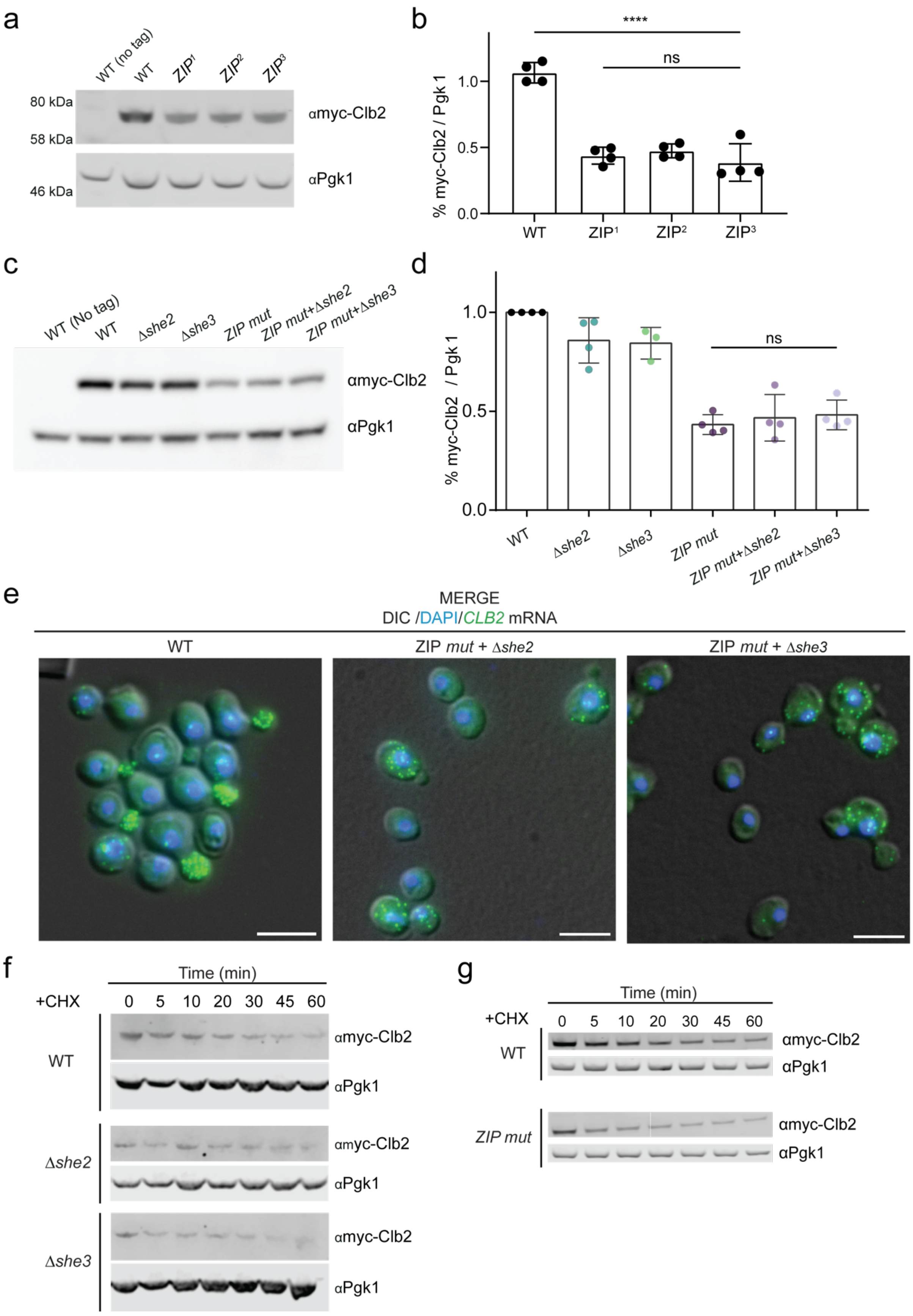

a
WT (no tag)
WT
ZIP¹
ZIP²
ZIP³
80 kDa
58 kDa
46 kDa
αmyc-Clb2
αPgk1
b
****
ns
% myc-Clb2 / Pgk 1
WT
ZIP¹
ZIP²
ZIP³
c
WT (No tag)
WT
Δshe2
Δshe3
ZIP mut
ZIP mut+Δshe2
ZIP mut+Δshe3
αmyc-Clb2
αPgk1
d
ns
% myc-Clb2 / Pgk 1
e
MERGE
DIC /DAPI/CLB2 mRNA
WT
ZIP mut + Δshe2
ZIP mut + Δshe3
f
Time (min)
+CHX
0 5 10 20 30 45 60
WT
Δshe2
Δshe3
αmyc-Clb2
αPgk1
g
Time (min)
+CHX
0 5 10 20 30 45 60
WT
ZIP mut
αmyc-Clb2
αPgk1

Supplementary Figure 5. Multiple ZIP code mutants show a reduction in Clb2 protein expression and *CLB2* mRNA mislocalization is not affecting protein stability.
(a) Western blot analysis using anti-myc antibody against Clb2 protein endogenously tagged with 5 myc tags in WT, ZIP^1, ZIP^2 and ZIP^3. First lane is the control untagged strain. Endogenous Pgk1 protein was used as loading control. **(b)** Quantifications of western blot in (a). Myc signal normalized to Pgk1 loading control. Protein levels relative to WT indicated. Mean ± SD from 4 replicates. **(c)** Western blot analysis using anti-myc antibody against Clb2 protein endogenously tagged with 5 myc tags in WT, *Δshe2*, *Δshe3*, ZIP mutant (ZIP^1), ZIP mutant (ZIP^1)+ *Δshe2,* ZIP mutant (ZIP^1)+ *Δshe3*. First lane is the control untagged strain. Endogenous Pgk1 protein was used as loading control. **(d)** Quantifications of western blot in (c). Myc signal normalized to Pgk1 loading control. Protein levels relative to WT indicated. Mean ± SD from 4 replicates. **(e)** smFISH WT, ZIP mutant + *Δshe2,* ZIP mutant + *Δshe3* strains. MERGE, maximal projections of *CLB2* smFISH (green) and DAPI (blue) merged to a single DIC picture (grey). Scale bars 5 µm. **(f)** Example of western blot of Clb2 protein stability assay in WT, *Δshe2* and *Δshe3* cells. Western blot was performed using an anti-myc antibody to target Myc-tagged Clb2 protein tagged in cells treated with 100 µg/ml cycloheximide (CHX) for 0, 5, 10, 20, 30, 45 and 60 minutes. Pgk1 protein was used as loading control. Quantifications are reported in Fig. 3e. **(g)** Example of western blot of Clb2 protein stability assay in WT and ZIP mutant cells. Western blot was performed using an anti-myc antibody to target Clb2 protein tagged with 5 myc tags in cells treated with 100 µg/ml cycloheximide for 0, 5, 10, 20, 30, 45 and 60 minutes. Pgk1 was used as loading control. Quantifications are reported in Fig. 3f. Source data are provided as a Source Data file.

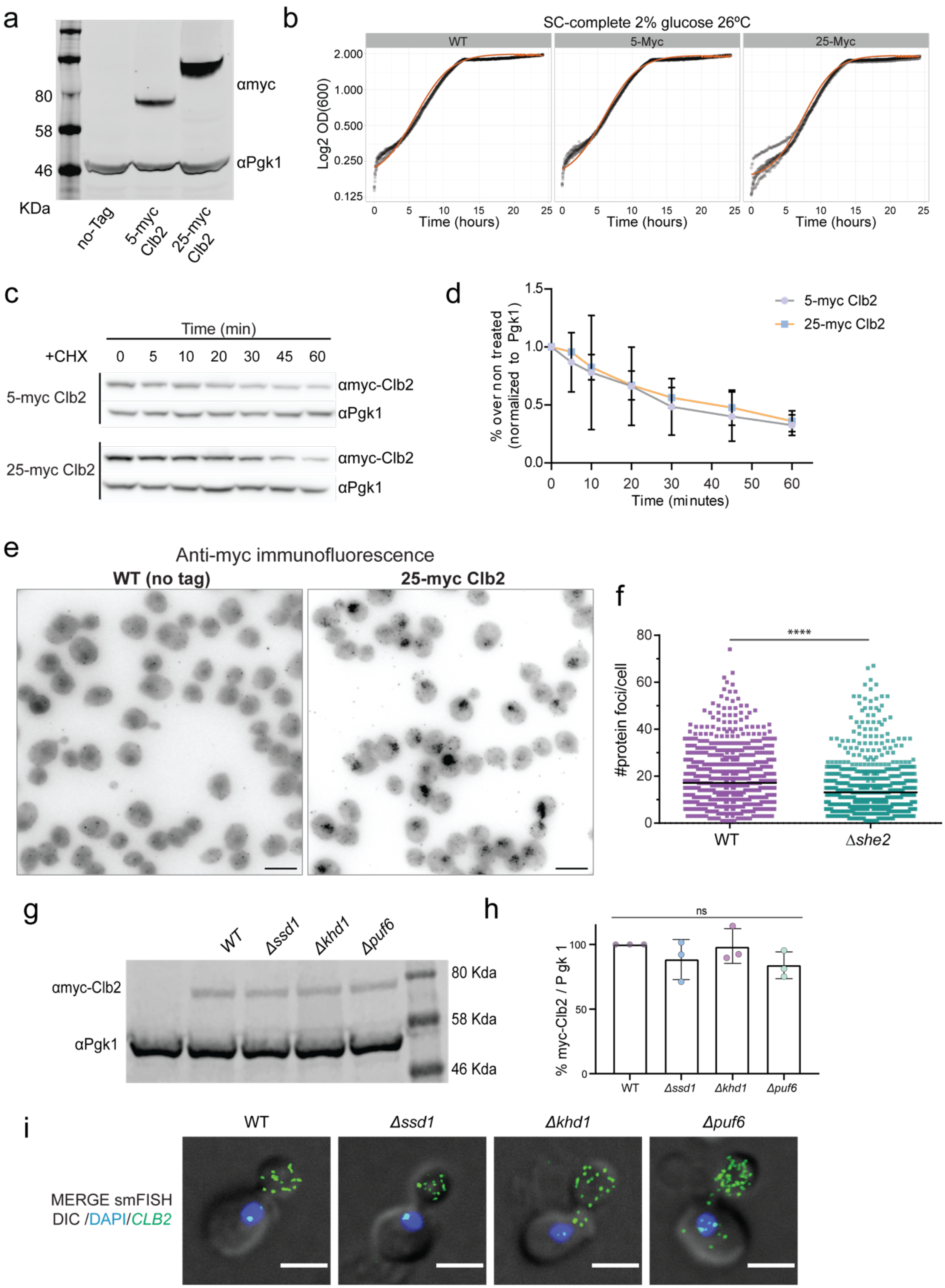

a
80
58
46
KDa
αmyc
αPgk1
no-Tag
5-myc Clb2
25-myc Clb2
b
SC-complete 2% glucose 26ºC
WT
5-Myc
25-Myc
Log2 OD(600)
Time (hours)
c
Time (min)
+CHX 0 5 10 20 30 45 60
5-myc Clb2
25-myc Clb2
αmyc-Clb2
αPgk1
d
% over non treated (normalized to Pgk1)
Time (minutes)
5-myc Clb2
25-myc Clb2
e
Anti-myc immunofluorescence
WT (no tag)
25-myc Clb2
f
#protein foci/cell
****
WT
Δshe2
g
WT
Δssd1
Δkhd1
Δpuf6
αmyc-Clb2
αPgk1
80 Kda
58 Kda
46 Kda
h
ns
% myc-Clb2 / Pgk 1
WT
Δssd1
Δkhd1
Δpuf6
i
WT
Δssd1
Δkhd1
Δpuf6
MERGE smFISH
DIC /DAPI/CLB2

Supplementary Figure 6. Known translation inhibitors are not involved in regulating Clb2 protein expression.

(a) Western blot analysis of *CLB2* myc tagged strains. Top row is the myc signal. Bottom row is the Pgk1 loading control. First lane is untagged cells. Second and third lanes are the Clb2 protein tagged with 5 and 25 myc tags, respectively. **(b)** Growth curves of strains with Myc-tagged Clb2 protein performed in Synthetic Complete media supplemented with 2% glucose, at 26°C. Growth curves are fitted to a logistic curve (red curve). **(c)** Example of western blot of Clb2 protein stability assay in 5-myc Clb2 and 25-myc Clb2 strains. Western blot was performed using an anti-myc antibody to detect Clb2 in cells treated with 100 µg/mL cycloheximide for 0, 5, 10, 20, 30, 45 and 60 minutes. Pgk1 was used as a loading control. **(d)** Quantifications of western blot shown in (c). The Clb2 signal was normalized to Pgk1 and to the time point 0 (non-treated). Black bars indicate mean ± SD from 3 independent experiments **(e)** Immunofluorescence using the anti-myc antibody in WT and 25-my Clb2 strains. A MAX projection is shown in gray scale. Scale bars 5 µm. **(f)** Quantifications of 25-myc-Clb2 immunofluorescence in WT and *Δshe2* strains, all cell cycle phases pulled together. Black bar indicates mean (WT=17.2±10.9 n=1099; *Δshe2*=13.1±10.9 n=1023). Statistical analysis: Mann-Whitney test P value <0.0001. **(g)** Western blot analysis using anti-myc antibody against Clb2 protein tagged with 5 myc tags in WT, *Δssd1, Δkhd1* and *Δpuf6* cells. Pgk1 protein was used as a loading control. First lane is untagged cells. **(h)** Quantification of (g). Mutant strain signal is normalized to WT signal. Each dot corresponds to one replicate experiment. Error bars indicate mean ± SD (WT= 100; Δssd1= 88.3±15.6; Δkhd1= 98.8±13.5; Δpuf6= 83.9±10.4). **(i)** Merge maximal projections of *CLB2* smFISH (green), DIC (gray), and DAPI (blue) in WT, *Δssd1, Δkhd1* and *Δpuf6* cells. Scale bars 3 µm. Source data are provided as a Source Data file.

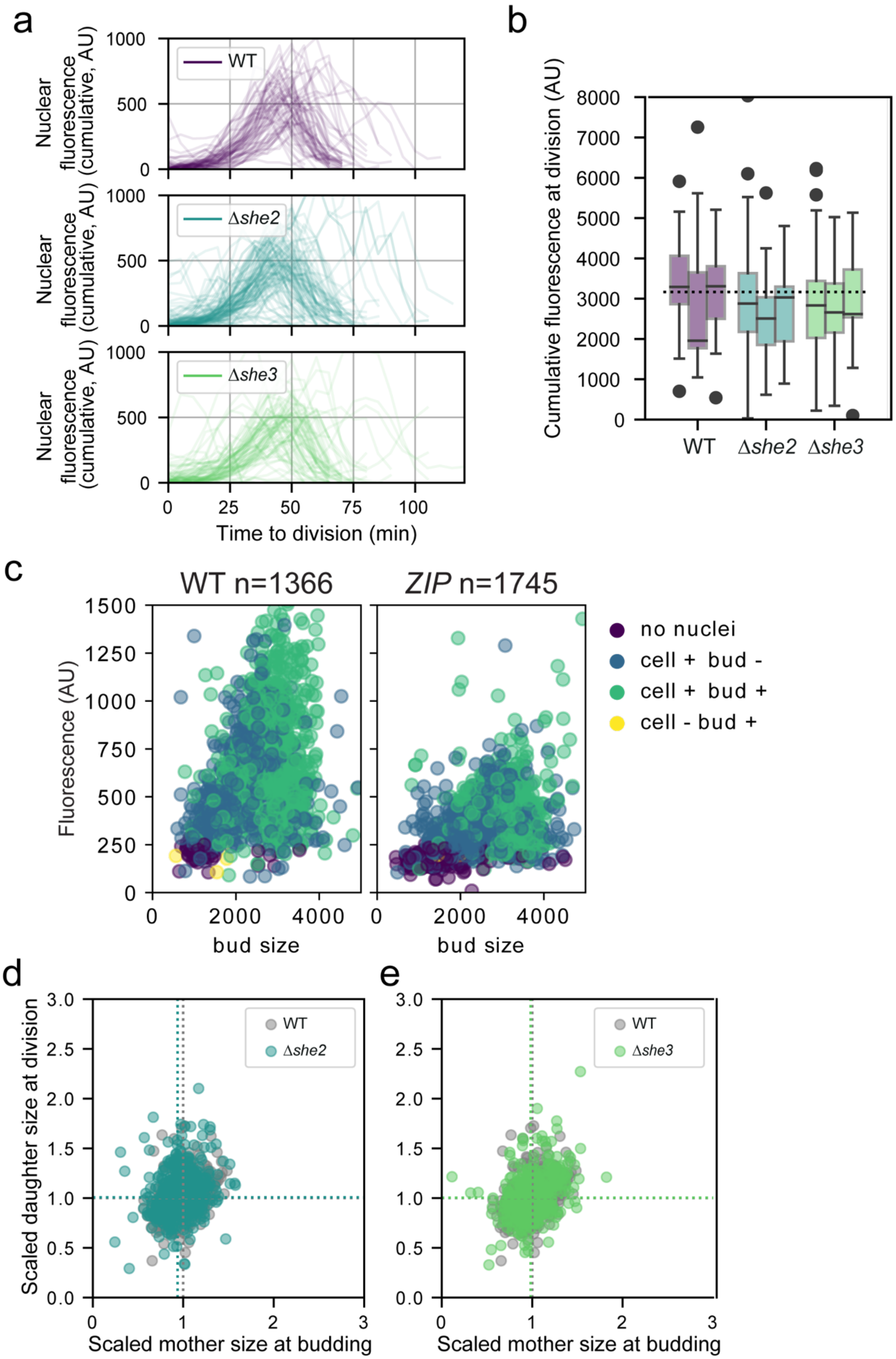
a
b
c
d
e
WT
Δshe2
Δshe3
Nuclear fluorescence (cumulative, AU)
Time to division (min)
Cumulative fluorescence at division (AU)
WT n=1366
ZIP n=1745
no nuclei
cell + bud -
cell + bud +
cell - bud +
Fluorescence (AU)
bud size
Scaled daughter size at division
Scaled mother size at budding

Supplementary Figure. 7: *Δshe* mutations have minor effects on cell size.
(a) Average fluorescence of Clb2-yeGFP of the 10% brightest pixels per cell as a proxy for nuclear fluorescence over time. Traces shown are recorded between budding and division for cells born in during the experiment and are pooled from three biological replicates. **(b)** boxplots showing the cumulative signal of the trajectories shown in (a) WT vs *Δshe2*: p=0.507, WT vs *Δshe3*: p=0.324. **(c)** nuclear fluorescence vs. bud size for all cells in the budded phase. Purple and blue are from pre-mitotic cells (no visible nucleus (purple), one visible nucleus (blue), green and yellow cells are post-mitotic and stray detections, respectively (green cells have elliptical nuclear signals or a detectable nucleus in both cell and bud compartment, yellow cells have a detectable nucleus in the bud compartment but not in the cell compartment. **(d)** Normalized scatter of scaled daughter size at division vs scaled mother size at budding for *Δshe2* n=598, vs WT (n=431), and **(e)** for *Δshe3,* (n=475) vs WT. Source data are provided as a Source Data file.

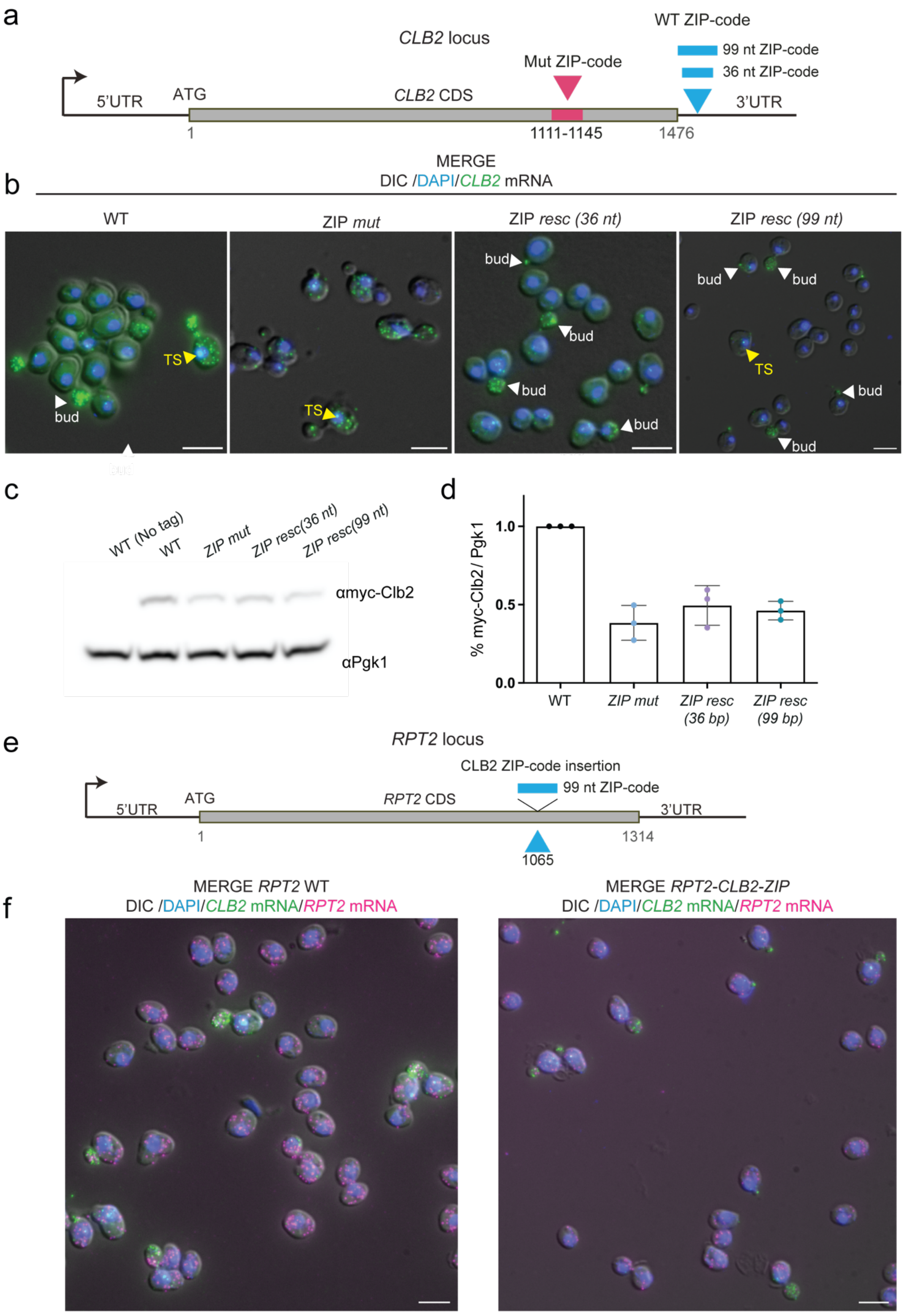
a
CLB2 locus
WT ZIP-code
99 nt ZIP-code
36 nt ZIP-code
Mut ZIP-code
5'UTR
ATG
CLB2 CDS
3'UTR
1
1111-1145
1476
b
MERGE
DIC /DAPI/CLB2 mRNA
WT
ZIP mut
ZIP resc (36 nt)
ZIP resc (99 nt)
TS
bud
c
WT (No tag)
WT
ZIP mut
ZIP resc(36 nt)
ZIP resc(99 nt)
αmyc-Clb2
αPgk1
d
% myc-Clb2/ Pgk1
1.0
0.5
0.0
WT
ZIP mut
ZIP resc (36 bp)
ZIP resc (99 bp)
e
RPT2 locus
CLB2 ZIP-code insertion
99 nt ZIP-code
5'UTR
ATG
RPT2 CDS
3'UTR
1
1314
1065
f
MERGE RPT2 WT
DIC /DAPI/CLB2 mRNA/RPT2 mRNA
MERGE RPT2-CLB2-ZIP
DIC /DAPI/CLB2 mRNA/RPT2 mRNA

Supplementary Figure 8. The *CLB2* ZIP code and its neighboring sequences promote gene expression, but are not sufficient to localize the *RPT2* mRNA
(a) Schematic of *CLB2* ZIP code rescue strains. The *CLB2* coding sequence (CDS) is shown in gray. The pink box represents the mutated ZIP code at nucleotides 1111-1145 (nucleotide number relative to START codon). The blue boxes represent the 36 nt or the 99 nt WT ZIP code variants inserted 41 nt after the stop codon in the 3'UTR of the endogenous *CLB2* gene. **(b)** smFISH in WT, ZIP code mutant and ZIP code rescue 36 nt and ZIP code rescue 99 nt strains. MERGE, maximal projections of *CLB2* smFISH (green) and DAPI (blue) merged to a single DIC picture (gray). Scale bars 5 µm. **(c)** Western blot analysis using anti-myc antibody against Clb2 protein endogenously tagged with 5 myc tags in WT, ZIP mutant and ZIP code rescue 36 nt and ZIP code rescue 99 nt strains. First lane is the control untagged strain. The endogenous Pgk1 protein was used as a loading control. **(d)** Quantification of (c). Mutant strain signal is normalized to WT signal. Each dot corresponds to one replicate experiment. Error bars indicate mean ± SD. **(e)** Schematic of *RPT2* gene modified with the *CLB2* ZIP code. The *RPT2* coding sequence has a length (1314 nt) comparable to the *CLB2* cds (1476 nt). The blue box represents the 99 nt *CLB2* ZIP code inserted in the *RPT2* CDS 1065 nt after the start codon, at a position resembling the *CLB2* ZIP code. Error bars represent mean +/- SD **(f)** smFISH in WT and *RPT2-CLB2-ZIP* strains. MERGE, maximal projections of *RPT2* mRNA smFISH (magenta), *CLB2* mRNA smFISH (green) and DAPI (blue) merged to a single DIC picture (gray). Scale bars 5 µm. Source data are provided as a Source Data file.

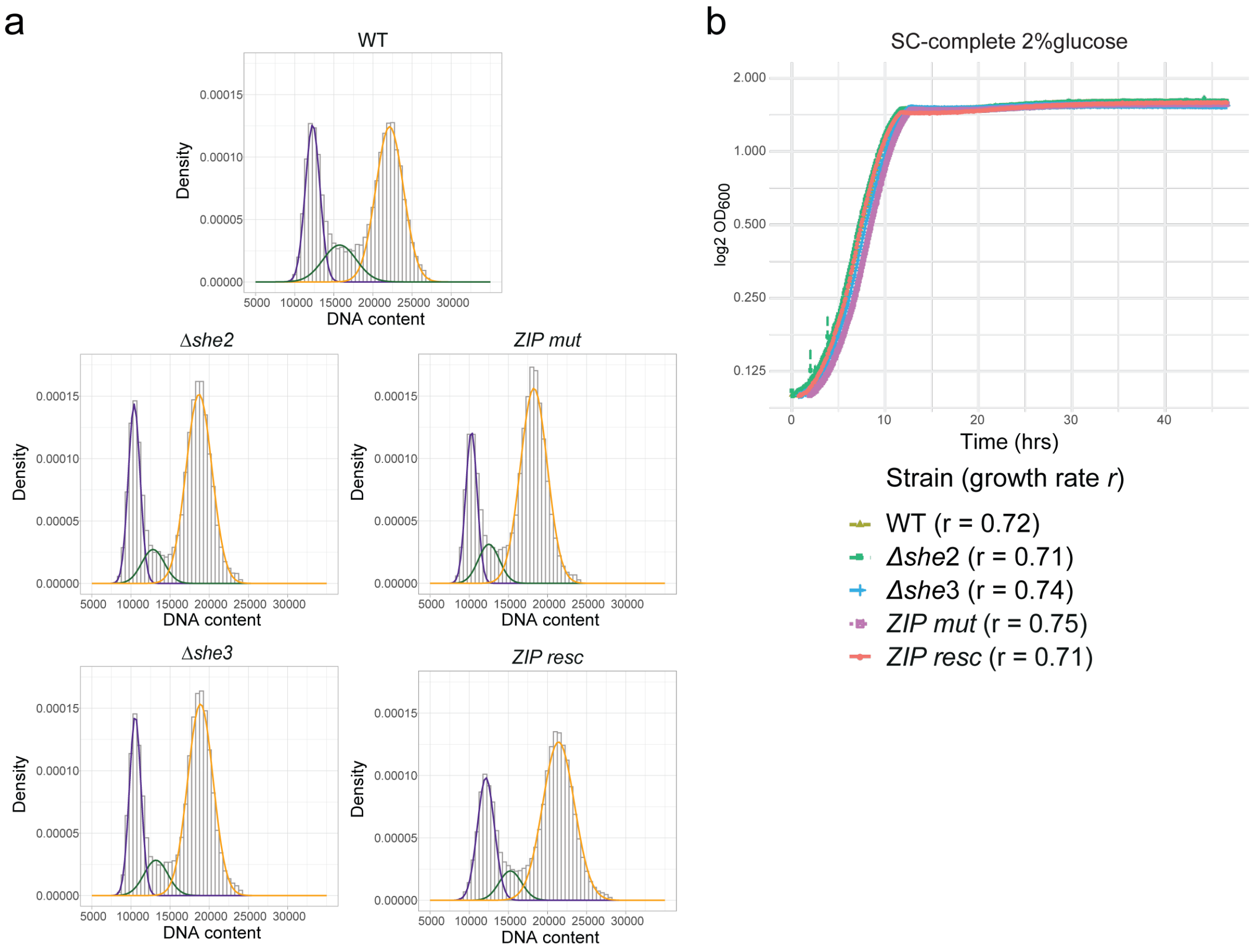

Supplementary Figure 9. The CLB2 localization mutants do not show significant growth rates changes while the ZIP mutant shows an increase in the G2/M population

(a) Cell cycle analysis by DNA content estimation with flow cytometry in in WT, *Δshe2*, *Δshe3*, ZIP code mutant and ZIP code rescue cells. Representative experiment showing the results of the mixed Gaussian fitting to estimate the three subpopulations, G1 (purple), S (green) and G2/M (yellow). **(b)** Growth curves of WT, *Δshe2*, *Δshe3*, ZIP code mutant and ZIP code rescue strains performed in Synthetic Complete medium supplemented with 2% glucose at 30°C. Source data are provided as a Source Data file.

Uncropped Western Blots

Related to Supplementary Figure 5

Figure S5a

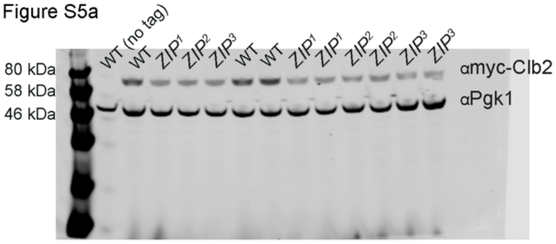

Figure S5c

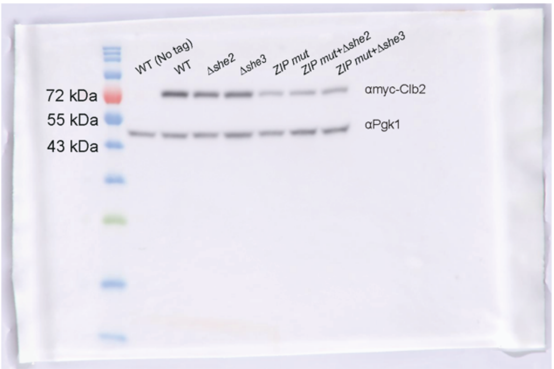

Figure S5f

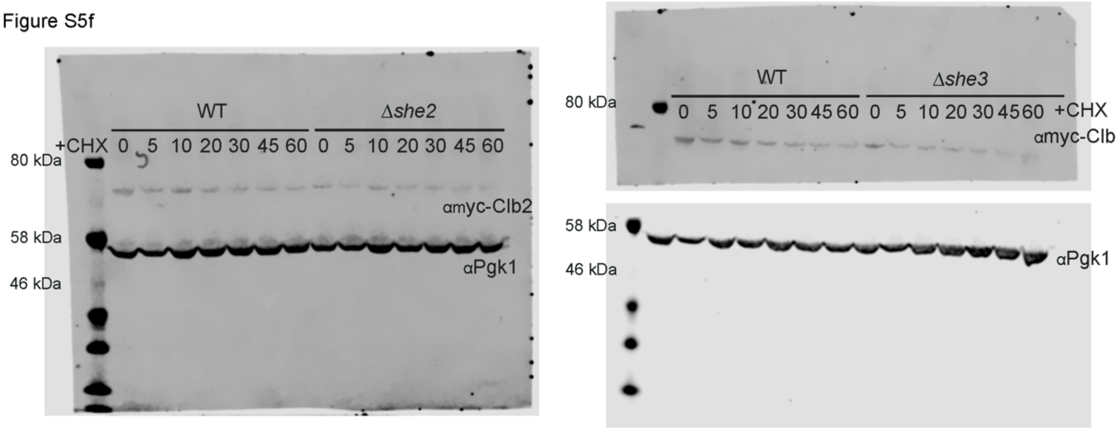

Figure S5g

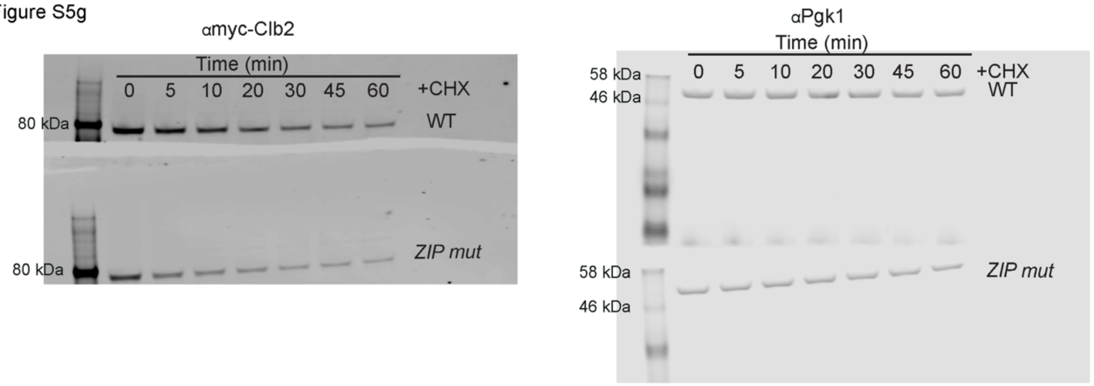

Related to Supplementary Figure 6

Figure S6a

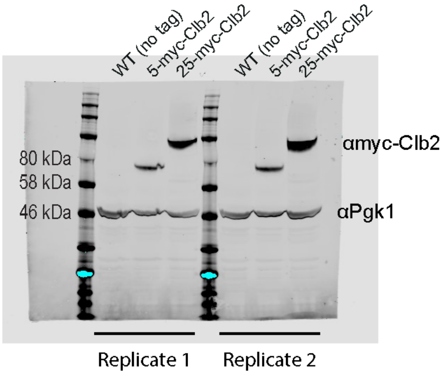

Figure S6g

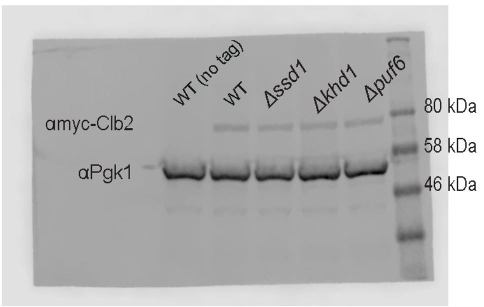

Related to Supplementary Figure 6c

Replicate 1

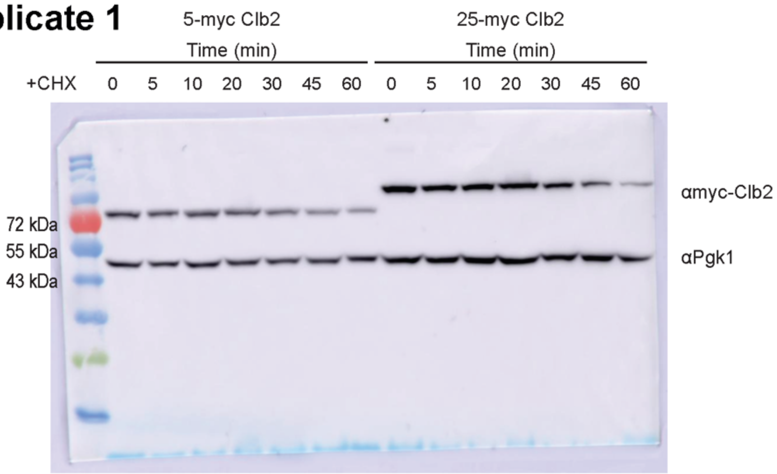

Replicate 2

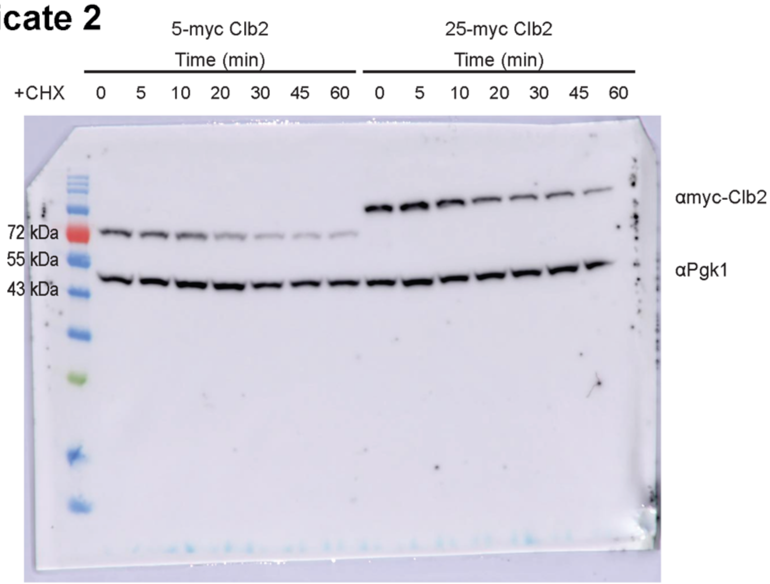

Replicate 3

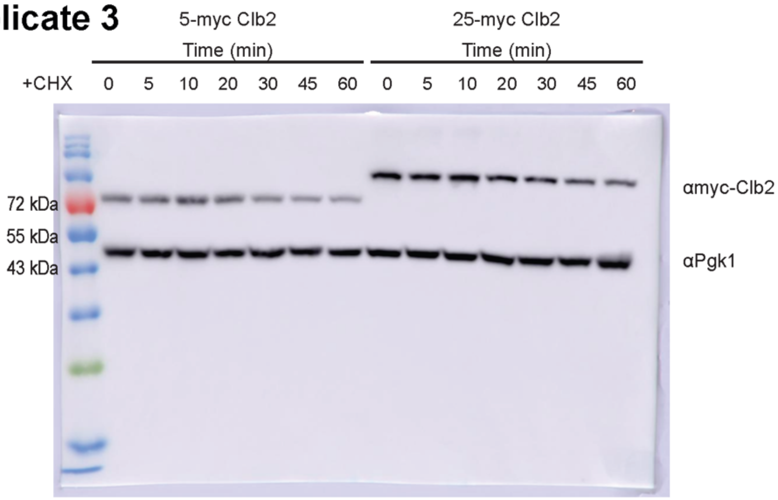

Related to Supplementary Figure 8

Figure 8c

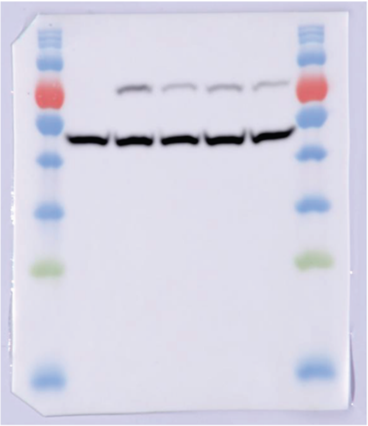

Supplement: Supplementary file 1 — Supplementary Information [file 41467_2025_66623_MOESM1_ESM.pdf]
