# Supplementary material for: Cyclin CLB2 mRNA localization and protein synthesis link cell cycle progression to bud growth
Source: Nat Commun. 2025 Nov 26;16:11654. doi: 10.1038/s41467-025-66623-w (PMC12749197; doi:10.1038/s41467-025-66623-w)
Supplement: Supplementary file 2 — Description of Additional Supplementary Files [file 41467_2025_66623_MOESM2_ESM.pdf]

Description of Additional Supplementary Files

File Name: Supplementary Data 1

Description: 1.Yeast strains List and genotype
2.Plasmids List
3.Oligonucleotides used for Plasmids and Yeast strains generation
4.smFISH Probes List and Sequences
5.ZIP codes and mutants

File name: Supplementary Movie 1

Description: CLB2 mRNA imaging in live cells throughout the cell cycle

CLB2 endogenously tagged with 24xMBSV6 to enable visualization of the mRNA (black) in live cells. The bud neck protein Cdc10-tdTomato is shown in magenta. Two-color imaging was performed by acquiring 13 Z-stacks every 0.5 µm, at two-minutes intervals and with exposure time of 50 ms. In video, at each time point, Z-stacks were max-projected. Scale bar 3 µm.

File name: Supplementary Movie 2

Description: CLB2 mRNA imaging in live cells at high frame-rate acquisition

CLB2 endogenously tagged with 24xMBSV6 to enable visualization of the mRNA (black) in live cells. The bud neck protein Cdc10-tdTomato is shown in magenta. A single plane was captured every 50 ms. Scale bar 2 µm.

File name: Supplementary Movie 3

Description: Clb2 protein imaging in WT cells

CLB2 endogenously tagged with yeGFP to enable visualization of the protein (green) in WT live cells. The bud neck protein mCherry-Cdc10 is shown in magenta. One single Z-stack was acquired every 5 minutes. Clb2-GFP was imaged with 200 ms exposure, mCherry-Cdc10 was imaged with 100 ms exposure and brightfield images were collected with 20 ms exposure. Scale bar 5 µm.

File name: Supplementary Movie 4

Description: Clb2 protein imaging in Δshe2 cells

CLB2 endogenously tagged with yeGFP to enable visualization of the protein (green) in Δshe2 live cells. The bud neck protein mCherry-Cdc10 is shown in magenta. One single Z-stack was acquired every 5 minutes. Clb2-GFP was imaged with 200 ms exposure, mCherry-Cdc10 was imaged with 100 ms exposure and brightfield images were collected with 20 ms exposure. Scale bar 5 µm.

File name: Supplementary Movie 5

Description: Clb2 protein imaging in Δshe3 cells

CLB2 endogenously tagged with yeGFP to enable visualization of the protein (green) in Δshe3 live cells. The bud neck protein mCherry-Cdc10 is shown in magenta. One single Z-stack was acquired every 5 minutes. Clb2-GFP was imaged with 200 ms exposure, mCherry-Cdc10 was imaged with 100 ms exposure and bright field images were collected with 20 ms exposure. Scale bar 5 μm.

File name: Supplementary Movie 6

Description: Clb2 protein imaging in ZIP-code mutant cells

CLB2 endogenously tagged with yeGFP to enable visualization of the protein (green) in ZIP-code mutant live cells. The bud neck protein mCherry-Cdc10 is shown in magenta. One single Z-stack was acquired every 5 minutes. Clb2-GFP was imaged with 200 ms exposure, mCherryCdc10 was imaged with 100 ms exposure and brightfield images were collected with 20 ms exposure. Scale bar 5 μm.
