# Supplementary material for: Cyclin CLB2 mRNA localization and protein synthesis link cell cycle progression to bud growth
Source: Nat Commun. 2025 Nov 26;16:11654. doi: 10.1038/s41467-025-66623-w (PMC12749197; doi:10.1038/s41467-025-66623-w)
Supplement: Supplementary file 10 — Reporting Summary [file 41467_2025_66623_MOESM10_ESM.pdf]

nature portfolio

Corresponding author(s): Evelina Tutucci

Last updated by author(s): Oct 25, 2025

Reporting Summary

Nature Portfolio wishes to improve the reproducibility of the work that we publish. This form provides structure for consistency and transparency in reporting. For further information on Nature Portfolio policies, see our Editorial Policies and the Editorial Policy Checklist.

Statistics

For all statistical analyses, confirm that the following items are present in the figure legend, table legend, main text, or Methods section.

n/a	Confirmed	
☐	☒	The exact sample size (*n*) for each experimental group/condition, given as a discrete number and unit of measurement
☐	☒	A statement on whether measurements were taken from distinct samples or whether the same sample was measured repeatedly
☐	☒	The statistical test(s) used AND whether they are one- or two-sided<br>*Only common tests should be described solely by name; describe more complex techniques in the Methods section.*
☒	☐	A description of all covariates tested
☐	☒	A description of any assumptions or corrections, such as tests of normality and adjustment for multiple comparisons
☐	☒	A full description of the statistical parameters including central tendency (e.g. means) or other basic estimates (e.g. regression coefficient) AND variation (e.g. standard deviation) or associated estimates of uncertainty (e.g. confidence intervals)
☐	☒	For null hypothesis testing, the test statistic (e.g. *F*, *t*, *r*) with confidence intervals, effect sizes, degrees of freedom and *P* value noted<br>*Give P values as exact values whenever suitable.*
☐	☒	For Bayesian analysis, information on the choice of priors and Markov chain Monte Carlo settings
☒	☐	For hierarchical and complex designs, identification of the appropriate level for tests and full reporting of outcomes
☐	☒	Estimates of effect sizes (e.g. Cohen's *d*, Pearson's *r*), indicating how they were calculated

Our web collection on statistics for biologists contains articles on many of the points above.

Software and code

Policy information about availability of computer code

Data collection	Flow cytometry: Beckman CytExpert 2.4.0.28<br>smFISH, smFISH-IF: Olympus - Cellsens V2<br>Live-cell imaging: Nikon NIS Elements 4.51<br>Western blotting: LITE software and Amersham software
Data analysis	live-cell imaging: python libraries hydra, pims, cellpose, pytorch, scikit-image, scikit-learn, astropy, photutils, pandas, numpy, matplotlib, scipy, networkx, napari, Fiji<br>smFISH, smFISH-IF: Matlab (Fishquant), Matematica.<br>Westernblots: Fiji<br>Flowcytometry: R<br>Data plotting: R, Phython, Graphpad Prism

For manuscripts utilizing custom algorithms or software that are central to the research but not yet described in published literature, software must be made available to editors and reviewers. We strongly encourage code deposition in a community repository (e.g. GitHub). See the Nature Portfolio guidelines for submitting code & software for further information.

Data

Policy information about availability of data

All manuscripts must include a data availability statement. This statement should provide the following information, where applicable:

- Accession codes, unique identifiers, or web links for publicly available datasets
- A description of any restrictions on data availability
- For clinical datasets or third party data, please ensure that the statement adheres to our policy

The raw data plotted in this manuscript can be found in the file 'Source Data' and in the Zenodo repository DOI: 10.5281/zenodo.16032630. In addition, the Resource table (Excel file) provides additional information about the reagents (i.e. plasmids, yeast strains, primers, smFISH probes) used in this study. Raw microscopy images analyzed in this study can be requested to the lead author.

Research involving human participants, their data, or biological material

Policy information about studies with human participants or human data. See also policy information about sex, gender (identity/presentation), and sexual orientation and race, ethnicity and racism.

Reporting on sex and gender	n/a
Reporting on race, ethnicity, or other socially relevant groupings	n/a
Population characteristics	n/a
Recruitment	n/a
Ethics oversight	n/a

Note that full information on the approval of the study protocol must also be provided in the manuscript.

Field-specific reporting

Please select the one below that is the best fit for your research. If you are not sure, read the appropriate sections before making your selection.

- [x] Life sciences
- [ ] Behavioural & social sciences
- [ ] Ecological, evolutionary & environmental sciences

For a reference copy of the document with all sections, see nature.com/documents/nr-reporting-summary-flat.pdf

Life sciences study design

All studies must disclose on these points even when the disclosure is negative.

Sample size	For sample size determination, we adhered to common practice in the field, striking a balance between practicality and robustness. Specifically, for smFISH data we collected enough images to be able to analyze about 1000 cells pooled from multiple biological replicates. For western blot we reported the averages of at least three biological replicates. Sample size single cell protein live imaging: sample size concerns either individual cells or trajectories of cells and is indicated in the figure or figure legend.
Data exclusions	live-cell imaging: G2 durations: trajectories were excluded based on average confidence assigned by a logistic classifier that was trained on shape of the neck compartment. Cells not captured by initial segmentation were excluded by default.
Replication	For all experiments we reported the results of independent biological replicates. The number of replicates vary depending on the experiment and it is reported in the figure legends.
Randomization	Randomization was not used in this study because the data collection is not influenced by sample allocation process. The analysis of the microscopy data was automated, therefore not affected by the sample order.
Blinding	Blinding was not used because the subjective analysis of the data was not used. Each experiment was analyzed using quantitative apporaches described in the methods, minimizing biased assessment.

Reporting for specific materials, systems and methods

We require information from authors about some types of materials, experimental systems and methods used in many studies. Here, indicate whether each material, system or method listed is relevant to your study. If you are not sure if a list item applies to your research, read the appropriate section before selecting a response.

Materials & experimental systems

n/a	Involved in the study
☐	☒ Antibodies
☒	☐ Eukaryotic cell lines
☒	☐ Palaeontology and archaeology
☐	☒ Animals and other organisms
☒	☐ Clinical data
☒	☐ Dual use research of concern
☒	☐ Plants

Methods

n/a	Involved in the study
☒	☐ ChIP-seq
☐	☒ Flow cytometry
☒	☐ MRI-based neuroimaging

Antibodies

Antibodies used: mouse anti-myc (Sigma SAB4700447), mouse anti-Pgk1 (Thermo Fisher scientific Prod# 459250)

Validation: For the anti-myc antibody, which was used to quantify a tagged version of the Clb2 protein, as a control we used a S. cerevisiae wild-type strain (BY4741) where the Clb2 protein was not myc-tagged. This control is presented in each of the western blots presented in this manuscript. The anti-Pgk1 antibody was used as a loading control. As a control, we used comassie staining of the acrylamide gels post -transfer, to verify equal loading for all the samples.

Animals and other research organisms

Policy information about studies involving animals; ARRIVE guidelines recommended for reporting animal research, and Sex and Gender in Research

Laboratory animals: n/a

Wild animals: n/a

Reporting on sex: n/a

Field-collected samples: n/a

Ethics oversight: n/a

Note that full information on the approval of the study protocol must also be provided in the manuscript.

Plants

Seed stocks: n/a

Novel plant genotypes: n/a

Authentication: n/a

Flow Cytometry

Plots

Confirm that:

☐ The axis labels state the marker and fluorochrome used (e.g. CD4-FITC).

☒ The axis scales are clearly visible. Include numbers along axes only for bottom left plot of group (a 'group' is an analysis of identical markers).

☐ All plots are contour plots with outliers or pseudocolor plots.

☒ A numerical value for number of cells or percentage (with statistics) is provided.

Methodology

Sample preparation: This is described in detail in the methods section

Instrument	Beckman Coulter CytoFLEX S Flow Cytometer (B2-R0-V2-Y2)
Software	Beckman CytExpert 2.4.0.28
Cell population abundance	50000 cells per sample
Gating strategy	Reported in R script that is deposited in zenodo: 10.5281/zenodo.16032630

☐ Tick this box to confirm that a figure exemplifying the gating strategy is provided in the Supplementary Information.
